# The Use of Oxygen/Air Blender during Transoral Laser Microsurgery with Supraglottic Manual Jet Ventilation: A Safe Approach

**DOI:** 10.1155/2023/5516988

**Published:** 2023-09-04

**Authors:** Sara Abu-Ghanem, James Cochran

**Affiliations:** ^1^Department of Otolaryngology, State University of New York (SUNY) Downstate Medical Center, Brooklyn, New York, USA; ^2^Division of Otolaryngology, Maimonides Medical Center, Brooklyn, New York, USA

## Abstract

**Background:**

Surgical fires are known, preventable, and devastating complications of transoral microlaryngeal laser surgery. Several guidelines have recommended maintaining the fraction of inspired oxygen concentration (FiO_2_) at or below 30% for open delivery cases. We hereby present our experience utilizing an air/oxygen gas mixing device (blender) attached to a supraglottic manual jet ventilator during transoral laser microlaryngeal surgery in three cases to control oxygen levels.

**Methods:**

Retrospective chart review of three cases and literature review.

**Results:**

Three patients underwent microlaryngeal laser surgery and balloon dilation for the management of subglottic stenosis. All three patients were successfully ventilated throughout the procedures, and no major complications occurred intraoperatively. Two of three patients demonstrated symptomatic and clinical improvement at the first follow-up.

**Conclusions:**

This report demonstrates the successful use of an oxygen/air blender to reduce FiO_2_ to fire-safe levels of less than 30% during laser surgery of the airway using jet ventilation.

## 1. Introduction

Surgical fires are rare but devastating events [1–3]. Airway fire is a known preventable complication of transoral laser microlaryngeal surgery that can lead to catastrophic outcomes for patients [4–7]. The initiation and propagation of a fire require an oxidizer (i.e., oxygen and nitrous oxide), an ignition or heat source (i.e., laser, “Bovie”), and a fuel (i.e., tissue, drapes, endotracheal tube (ETT), and gauze) [8]. In airway cases, the flammable source is most commonly the ETT [4]. However, the risk of fire also exists in open systems when increased oxygen concentration and high energy sources are present in proximity [9–12]. Organic material such as carbonized/charred tissue or the laser smoke itself provides another readily flammable source [9, 13].

A key measure to prevent airway fires is to maintain the fraction of inspired oxygen concentration (FiO_2_) delivered to the patient at the minimum necessary to avoid hypoxia. Given the increased fire risk related to oxygen, the Joint Commission (Oakbrook Terrace, Illinois) and the Emergency Care Research Institute recommend the use of air or FiO_2_ less than or equal to 30% for open delivery cases [8]. Jet ventilation (JV) has become a popular ventilation approach for microlaryngeal surgery, allowing for endoscopic intervention without the interference of an ETT [14]. JV relies on the application of gas portions under high pressure through an unblocked catheter into the airway, which is open to the ambient air. Exhalation in JV is passive, and adequate gas exchange is heavily reliant on modification of respiratory rate, driving pressure, oxygen concentration, and inspiration time. It can be delivered in an infraglottic, supraglottic, transtracheal, or transluminal manner, with the use of an automated or manual (hand-held) device [15]. In both automated and manual JV, 100% oxygen delivery is possible, but many automated jet ventilators have blenders to mix oxygen and air to reduce the FiO_2_, yet these are not readily available in every center. In this report, we describe our experience with an external oxygen/air blender in reducing FiO_2_ delivered by manual JV in three cases of transoral microlaryngeal laser surgery.

## 2. Case Description

A retrospective chart review was conducted of the medical records of adult patients undergoing supraglottic JV for microdirect laryngoscopy (MDL) procedures using an oxygen/air blender between June 2020 and June 2021. Ethical approval for this study was not required. Three patients were identified in which the approach was used. Patient characteristics and intraoperative findings are summarized in Table 1.

**Figure 1:**
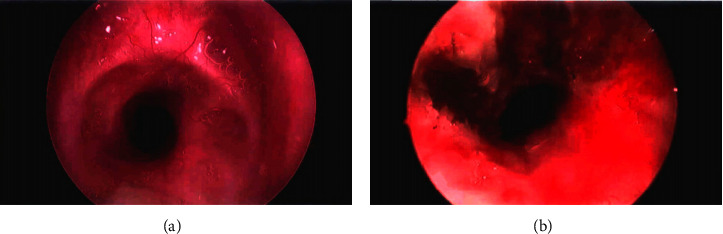
Preoperative (a) and intraoperative (b) images of stenotic subglottic area (patient #1).

- *Patient 1*. A 50-year-old female patient presented with a complicated medical history including left ventricular assist device implantation. The patient presented with grade III subglottic stenosis (SGS) of ∼80% narrowing secondary to prolonged intubation. The patient underwent MDL with radial laser incisions of stenosis using potassium-titanyl-phosphate (KTP) laser, balloon dilation, and steroid injection. The patient was ventilated with a jet ventilator attached to an oxygen/air blender (MaxBlend, Maxtec, USA) capable of providing an FiO_2_ between 21 and 100%. Intraoperative images captured with a rigid 0-degree telescope passed through the glottis are shown in Figures 1(a) and 1(b). The patient required intermittent brief oral intubation via Dedo laryngoscope with a 5-0 microlaryngoscopy tube (MLT) after developing significant bradycardia (heart rate <30/min) during balloon dilation. She was extubated successfully upon awakening from general anesthesia at the end of the procedure. No other complications, such as pneumothorax, aspiration, laryngospasm, or fire-induced injury, developed intraoperatively. The patient was discharged after two-day admission and was found to have a symptomatic improvement in breathing, with in-office nasal tracheoscopy (TNT) demonstrating improvement in airway narrowing to <50% at 2 months postoperatively.
- *Patient 2*. A 63-year-old male patient with a history of smoking, obesity, and COVID-19 pneumonia with respiratory failure requiring intubation and tracheostomy presented to the clinic with dysphonia and increasing dyspnea one month after tracheostomy decannulation. The patient was found to have grade III SGS on in-office TNT with at least 75% subglottic narrowing and glottic narrowing due to limited abduction of true vocal folds (VFs) and significant swelling consistent with polypoid corditis. He subsequently underwent MDL, with KTP laser incisions of stenosis and ablation of true VFs polypoid tissue. Supraglottic jet ventilation was used with the addition of an air/oxygen blender during periods of laser activation as described previously. The patient was briefly intubated with a 5-0 MLT intraoperatively due to a period of oxygen desaturation <90% but was successfully extubated after termination of general anesthesia and was monitored overnight. The patient had a complicated postoperative course, requiring tracheostomy due to continued stridor and glottic narrowing 2 days postinitial MDL. The patient remained cannulated at the follow-up visit one month postoperatively, with in-office TNT at that time demonstrating multilevel airway narrowing and minimal glottic opening with patent tracheal cannula.
- *Patient 3*. A 24-year-old female presented with progressively worsening shortness of breath over 1 year and was found to have grade II SGS with 50–60% airway narrowing. The patient underwent MDL with radial laser cuts of stenosis using CO_2_ laser, balloon dilation, steroid injection, and biopsy under supraglottic jet ventilation attached to an oxygen/air blender as described previously. There were no complications, and after overnight monitoring, the patient was discharged one day postoperatively with improved respiratory status. Two-week follow-up revealed symptomatic improvement in breathing with minimal dysphonia and dysphagia. In-office TNT showed a widely patent subglottis and less than 25% airway narrowing 3 months postsurgery.

All three patients underwent MDL with radial laser cuts, balloon dilation, and steroid injections for the management of SGS. KTP laser was chosen when CO_2_ laser was not available. Jet ventilation was accomplished under suspension laryngoscopy in all patients without difficulty. An oxygen blender (MaxBlend, Maxtec, USA), connected to the jet ventilator via high-pressure noncollapsible oxygen tubing, was utilized to successfully reduce the source oxygen to less than 30% FiO_2_ during laser activation in the airway. No airway fire events occurred throughout the entire duration of all procedures. Two patients (#1 and #2) required brief intubation with 5-0 MLT intraoperatively but were extubated successfully by the end of the procedure. Two patients (#1 and #3) experienced clinical and symptomatic improvement at 2-week follow-up. Patient #2 had a complicated postoperative course necessitating tracheostomy and continued to have multilevel airway stenosis with minimal improvement in glottic opening at follow-up.

## 3. Discussion

Surgical fires in otolaryngology continue to be widely reported in the literature. A survey of otolaryngologists conducted by Smith and Roy revealed that 27% of fires occurred during endoscopic airway surgery, 24% during oropharyngeal surgery, 23% during cutaneous surgery of the head and neck, and 18% during tracheostomy [16]. Day et al. reviewed existing literature in 2017 and found that 97% of reported otolaryngologic fire cases involved FiO_2_ levels of greater than 30% [4].

Based on our extensive review of the literature, we discovered limited data regarding the actual fire risk associated with open oxygen delivery systems and specifically jet ventilation in endoscopic laryngeal laser surgery. Generally, JV is considered safe compared to closed oxygen delivery systems (with ETT); however, it remains unclear what the expected FiO_2_ is with JV as this is multifactorial [15, 17]. The FiO_2_ can change depending on the technique (low- versus high-flow JV), mode of delivery (supraglottic, subglottic, and transtracheal), oxygen concentration, laser power, use of nasal cannula, or use of drapes that can cause an enriched O_2_ environment.

Thus, it continues to be necessary to reduce FiO_2_ delivered to the JV to fire-safe levels (e.g., 30%) if JV is to be used in the presence of fire hazards as lasers such as potassium-titanyl-phosphate (KTP) or CO_2_. Some modern automated jet ventilators allow for FiO_2_ selection (e.g., the Monsoon III, Acutronic® Medical Systems, Switzerland). In our experience, these automated ventilators are not readily available. Oxygen/air blenders are widely available and can be easily adapted for use with most existing jet ventilators. We adopted this approach to simultaneously meet patient safety standards and minimize costs by incorporating widely available technology.

This series adds to the existing literature by reporting on the effective use of an oxygen/air blender attached to a manual jet ventilator to allow for the safe activation of lasers in the airway even in the presence of high-flow oxygen. To our knowledge, this is the only published record of the use of an oxygen/air blender in this manner.

## 4. Conclusion

This report of three cases demonstrates the successful and safe use of an oxygen/air blender to reduce FiO_2_ to fire-safe levels during laser surgery of the airway using manual jet ventilation. Further studies and models are needed to investigate the fire risks in jet ventilation and open oxygen delivery systems in transoral laser surgery.

## Figures and Tables

**Table 1:** Preoperative data and operative data.

Patient	#1	#2	#3
*Preoperative data*			
Age (y)	50	63	24
Gender	F	M	F
Diagnosis	Subglottic stenosis	Subglottic stenosis Polypoid corditis	Subglottic stenosis
Etiology	Prolonged intubation	Prolonged intubation Post-tracheostomy	Idiopathic

*Operative data*			
Surgical interventions	MDL with KTP laser radial cuts, balloon dilation, and steroid injection	MDL with KTP laser radial cuts, balloon dilation, and steroid injection	MDL with CO_2_ laser radial cuts, balloon dilation, and steroid injection
Laser setting	KTP continuous mode 10 W	KTP continuous mode 5 W (stenosis) and 2 W (VFs ablation)	CO_2_ continuous mode 5 W
Complications^†^	Intubation	Intubation	None

CO_2_, carbon dioxide; FiO_2_, fraction of inspired oxygen concentration; KTP, potassium-titanyl-phosphate; MDL, microdirect laryngoscopy; VFs, vocal folds. ^†^Major intraoperative complications assess included unplanned intubation and airway fire or ignition.

## Data Availability

The data supporting the current study are available from the corresponding author upon request.
